# Electrocoagulation/flotation process for removing copper from an aqueous environment

**DOI:** 10.1038/s41598-023-40512-y

**Published:** 2023-08-16

**Authors:** Giti Kashi

**Affiliations:** 1 Department of Environmental Health Engineering, Faculty of Health, Tehran Medical Sciences Branch, Islamic Azad University, Khaghani St., Shariati Ave, Tehran, Iran; 2 Water Purification Research Center, Tehran Medical Sciences, Islamic Azad University, Tehran, Iran

**Keywords:** Environmental sciences, Chemistry

## Abstract

The presence of copper in aqueous environments such as drinking water has led to several environmental effects, such as flavor and odor. The increase in Cu levels in ground and surface water has been mainly attributed to anthropogenic and natural sources. Consequently, this applied-analytical study aimed to investigate copper removal from urban drinking water through batch reactor electrocoagulation/flotation (ECF) with aluminum electrodes. The copper removal efficiency was evaluated under various operating conditions of current density (0.8–2.4 mA/cm^2^), initial concentration (1–100 mg/L), pH (3.5–10.5), and time (10–30 min). Cu was determined using the method outlined in the standard procedures (3500-Cu B at 4571 nm). The results indicated that increasing the current density from 0.8 to 2.4 mA/cm^2^ and the reaction time from 10 to 30 min improved Cu^+2^ removal efficiency (from 95 to 100%). In addition, the results demonstrated that Cu^+2^ reduction is 100% with an initial concentration of 100 mg/L, a pH of 7.5, a reaction time of 30 min, and an anode current density of 2.4 mA/cm^2^. The Taguchi method results for copper removal efficiency show that reaction time is the most significant variable. Furthermore, Cu removal kinetics models in an ECF reactor are second-order (R^2^ > 0.92). The Cu removal in the ECF reactor is due to redox and adsorption. Moreover, the operational costs of Cu treatment with Al electrode pairs are estimated to range from 8857 and 9636 Rial/kg of Cu removed. Thus, it can be concluded that the ECF process is very efficient in removing Cu from aqueous environments under optimum conditions.

## Introduction

Copper (Cu) is a ductile metal with extremely high electrical and thermal conductivity. Cu is an essential trace mineral for all living organisms because it is a key constituent of the respiratory enzyme complex cytochrome oxidase. The Cu element exists in Cu^+1^ and Cu^+2^ forms^1,2^. Cu is present in the liver, the muscle, and the bone. Cu compounds are currently utilized as bacteriostatic substances, fungicides, and wood preservatives. Furthermore, copper sulfate (CuSO_4_) is widely employed as an algicide in aquatic environments^3^, where the high concentration of Cu in treated drinking water causes adverse health effects such as anemia, eye and skin irritation, and damage to the human brain and heart organs^4^.

Various copper compounds are used in tumor treatment^5^. In addition, a known association has been evidenced between abnormal Cu serum levels and Alzheimer's disease (AD)^6^. The US Environmental Protection Agency (EPA) states that the maximum concentration level (MCL) for copper in drinking water is 1.3 mg/L^7^. Cu and Cu enzymes affect energy metabolism, oxidative detoxification, and mitochondrial respiration^8^, where Cu and other micronutrients, such as iron, are essential for preventing AD^9^. Furthermore, anthropogenic (wire and cable, electronics and related devices, architecture, antimicrobial applications, wood manufacturing, industrial, mining, and agricultural activities, and sewage discharge) and natural (corrosion of household plumbing systems, rock weathering, rock and soil erosion, and atmospheric deposition) sources account for the majority of the increase in Cu levels in ground and surface water^10^. Due to its negative effects on human health and aquatic ecosystems, Cu is particularly considered in industrial wastewater treatment, where membrane separation, ion exchange, chemical precipitation, electrochemistry, adsorption, and biotechnology are among the applications^11^.

According to a systematic review conducted in Iran, the Cu concentration in drinking water exceeds permissible limits in 7.69% of the performed studies^12^. Cu concentration in 8 samples out of 58 samples are above the allowable limit (2.99 mg/L) in the drinking water sources of Karaj city, Iran^13^. According to the research conducted in 6 storm water ponds in Florida, the concentration of copper in sediments is several times that of water^14^. The important inputs of Cu to freshwater are natural sources (3.7 ktpa), agriculture (1.8 ktpa), and runoff (1.8 ktpa) in European Union^15^. The range of dissolved copper concentration is 6.4–45.4 nM in coastal and estuarine waters in water from a high industrialized and urban coastal system^16^.

Conventional processes for heavy metal removal such as Cu include adsorption (such as tobacco stem biochar), biosorption (such as Moringa oleifera seed biomass), ion exchange (tert-butyl 2-(methylamino)-N-acetic acid functionalized chelating resin), chemical precipitation (such as lime, soda ash, sodium sulfide), electrochemical, electrodeposition, membrane filtration (such as polyvinylamine by polymer-enhanced ultrafiltration and flocculation) methods^17–23^. The increased production of chemical sludge and the high stability of chemical precipitation are two of its disadvantages^10^. According to the research conducted in 6 rainwater ponds in Florida, the concentration of copper in sediments is several times that of waterTherefore, the Cu removal method must be novel and cost-effective, whereas recent years have seen the implementation of advanced oxidation processes (AOPs) such as combined electrocoagulation and flotation (ECF) for the treatment of wastewater contaminated with salicylic acid^24^.

Although in the electrocoagulation (EC) process with alternating pulse current, electricity consumption and electrode consumption for coagulant production have not been completely eliminated as weak points of the process. However, these disadvantages have been mitigated compared to the coagulation process and EC with direct current. By electrolyzing a consumed anode electrode, the ECF method generates coagulating agents. The ECF process involves the use of electric current and air bubbles in order to electrolyze, flocculate, and float contaminants without having to add coagulations. Efficiency of EC process is enhanced with external aeration^25^. EC aeration system has more efficiency than EC unit due to producing homogenous conditions and enhancing oxidation reaction. Effective factors influencing the performance of EC and ECF include electrode geometry, electrode material, geometry current density, water quality, and turbidity concentration^26^. The advantages of removing Cu by the ECF process are that no chemical agents are required, the pollutant in the water is removed by gas bubbles generated during the process, and the treatment is clean because no anion, other than hydroxide, is added (OH^−1^). This technique is a promising alternative to chemical coagulation due to not using chemical substances. The following equations illustrate this ECF mechanism involving aluminum anode and cathode electrodes and this process leads to the removal of Cu through the three mechanisms of electrode oxidation, the production of gas bubbles, and the processes of floating and settling the formed clots:

1 $${\text{Anode:}}{\text{ 2OH}}^{ - } \to {\text{O}}_{{2}} + {\text{ 2H}}^{ + } + {\text{ 4e}}^{ - } \left( {{\text{E}}^{0} \left( {{\text{O}}_{{2}} /{\text{OH}}^{ - } } \right) \, = \, 0.{4}0\;{\text{V}}} \right)$$

2 $${\text{Anode:}}{\text{ 2H}}_{{2}} {\text{O}} \to {\text{4H}}^{ + } + {\text{ 4e}}^{ - } + {\text{ O}}_{{2}} \left( {{\text{E}}^{0} \left( {{\text{O}}_{{2}} /{\text{H}}_{{2}} {\text{O}}} \right) \, = { 1}.{23}\;{\text{V}}} \right)$$

3 $${\text{Anode:}}{\text{ Al}} \to {\text{Al}}^{{{3} + }} + {\text{ 3e}}^{ - } \left( {{\text{E}}^{0} \left( {{\text{Al}}^{{{3} + }} /{\text{Al}}} \right) \, = \, - { 1}.{66}\;{\text{V}}} \right)$$

4 $${\text{Cathode}}:{\text{ 2H}}^{ + } + {\text{ 2e}}^{ - } \to {\text{H}}_{{2}} \left( {{\text{E}}^{0} \left( {{\text{2H}}^{ + } /{\text{H}}_{{2}} } \right) \, = \, 0.00\;{\text{V}}} \right)$$

5 $${\text{Cathode:}}{\text{2H}}_{{2}} {\text{O }} + {\text{ 2e}}^{ - } \to {\text{H}}_{{2}} + {\text{ 2OH}}^{ - } ({\text{E}}^{0} \left( {{\text{2H}}^{ + } /{\text{H}}_{{2}} {\text{O}}} \right) = - \, 0.{83}\;{\text{V}})$$

6 $${\text{O}}_{{2}} + {\text{ 2H}}^{ + } + {\text{ 4e}}^{ - } \to {\text{2H}}_{{2}} {\text{O }}({\text{E}}^{0} \left( {{\text{O}}_{{2}} /{\text{H}}_{{2}} {\text{O}}} \right) \, = { 1}.{23}\;{\text{V}})$$

7 $${\text{O}}_{{2}} + {\text{ 2H}}_{{2}} {\text{O }} + {\text{ 4e}}^{ - } \to {\text{4OH}}^{ - } ({\text{E}}^{0} \left( {{\text{O}}_{{2}} /{\text{OH}}^{ - } } \right) \, = \, 0.{4}0\;{\text{V}})$$

8 $${\text{Cu}}^{{{2} + }} + {\text{ 2e}}^{ - } \to {\text{Cu }}\left( {{\text{E}}^{0} \left( {{\text{Cu}}^{{{2} + }} /{\text{ Cu}}} \right) \, = \, 0.{34}0\;{\text{V}}} \right)$$

Based on the research history, ECF process to remove Cu is done for the first time in Iran. The Cu value in drinking water sources exceeds permissible limits in some parts of Iran country. From the aspects of innovation, similarities and differences of this research with other researches, it is possible to examine various variables in a research such as examining the concentration of copper in different ranges (1–100 mg/L), optimizing the process, examining the kinetics of the process, examining the mechanism of process, cost calculation and, examining the electrode surface mentioned. This study sought to remove Cu from drinking water using an ECF batch reactor with aluminum (Al) electrodes. The variables investigated were Cu concentration, current density, pH, and reaction time.

## Materials and Methods

### Preparation of electrodes

The weight of the electrode was measured after washing with distilled water. In order to remove impurities from the surface of the electrodes, the electrode was pre-treated by being polished with abrasive paper, washed with detergent and tap water, and rinsed with deionized water. The cleaned electrode was subsequently dried before being submerged in water in the reactor^27^.

### Experimental Setup

**Figure 1:**
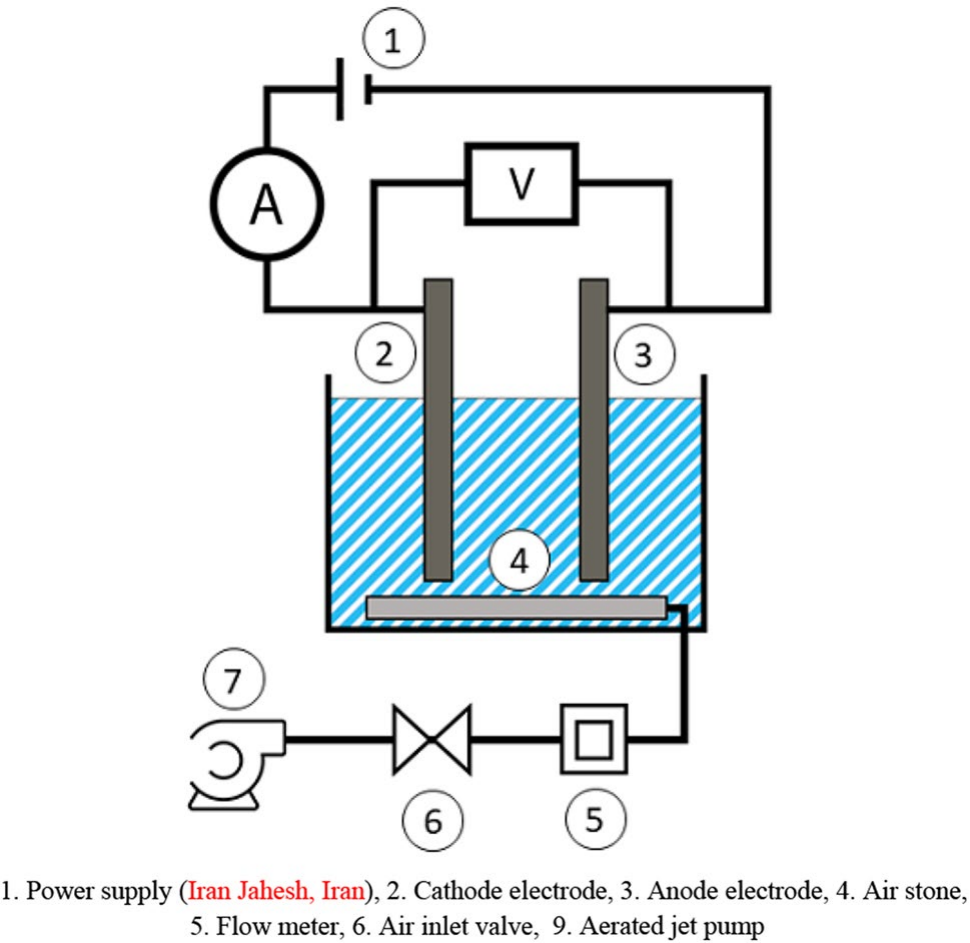
The batch ECF.

The batch ECF with monopole arrangement was a 10 × 6 × 6 cm (360 mL) glass vessel (Fig. 1). Al materials and Al were utilized as anode and cathode electrodes, respectively. Each electrode had an active surface area of 36 cm^2^ (9 × 4 × 0.1 cm). The distance between electrodes was adjusted to 2 cm. To determine the and effect of electrolysis on the Cu removal process, samples were exposed to varying Cu (Merck, Germany) concentrations (1–100 mg/L^−1^), current densities (0.8–2.4 mA/cm^2^), pH (approx. 3.5–10.5), and reaction times (10–30 min). Water samples were aerated using an air stone and an aerated jet pump (RS. Model 610, China). For each test, 200 mL of water samples were poured into the reactor. All tests were conducted at 20 °C in the laboratory. Chloride acid and sodium hydroxide solutions (0.1 N) (Merck, Germany) were used for pH adjustment.

### Analytical methods

All tests were performed in triplicate, and the mean data values were reported. The Cu oxide reduction potential (ORP), pH, and temperature of the water samples were determined after using ECF using a spectrophotometer (DR 5000, Hack, USA), an ORP meter (CG, Malaysia), and a pH meter (Hack, USA), respectively. Notably, Cu values were determined using prevalent standard procedures (3500-Cu B at 4571 nm)^28^.

The Cu removal percentage was calculated using Eq. (9)^29^:

9 $$R{ }\left( {\text{\% }} \right) = \left( {\frac{{C_{0} - C_{t} }}{{C_{0} }}} \right) \times 100$$

where ($R \left(\mathrm{\%}\right)$) denotes the percentage of Cu, and (${C}_{0}$ and ${C}_{t}$ in mg/L) represents the Cu value before and after treatment.

The operational cost required for Cu removal was calculated through Eq. (10)^29^:

10 $$Operational\, cost = C_{energy} + C_{electrode}$$

where (C_operational_ in Rial kWh per kg of Cu removed) denotes operational cost, ($C_{energy}$ in kWh/kg) represents the consumed electrical energy cost, and ($C_{electrode}$ in Rial per kg of Cu removed) is the consumed electrode cost.

The electrical energy required for Cu removal was calculated by Eq. (11):

11 $$EE{ }\left( {\frac{kWh}{{kg}}Cu\,removed{\text{\% }}} \right) = \left( {\frac{{VIt{ } \times 1000}}{{60{ } \times (C_{t0} - C_{t} }}} \right)$$

where $EE{ }\left( {\frac{kWh}{{kg}}Cu\,removed{\text{\% }}} \right)$ denotes the consumed electrical energy cost, $V$ represents the electrical voltage, (I, A) is the electrical current, and $t$ (min) is time.

Electrodes were rinsed with distilled water for 1 min after conducting all tests resulted in removing of deposits from the surface of the electrodes.

Kinetics reaction models were calculated through Eqs. (12) and (13)^30,31^:

12 $$\ln C_{t} = \ln c_{t0} - K_{1} t$$

13 $$\frac{1}{{C_{t} }} = K_{2} t + 1/C_{t0}$$

where $C_{0} { }$ and $C_{t}$ denote the concentration of Cu at the beginning and after time $t$ of the reaction, respectively, $K_{1}$ and $K_{2}$ represent the first and second-order reaction constants, respectively.

The slopes of the plots $\ln C_{t}$ versus $t$ and $\frac{1}{{C_{t} }}$ versus $t$ were used to determine the values of K_1_ and K_2_.

In addition, the Taguchi model was utilized to represent the relationship between four operational variables and three levels of removal efficiency^32^.

## Results

### Effect of Cu concentration

**Figure 2:**
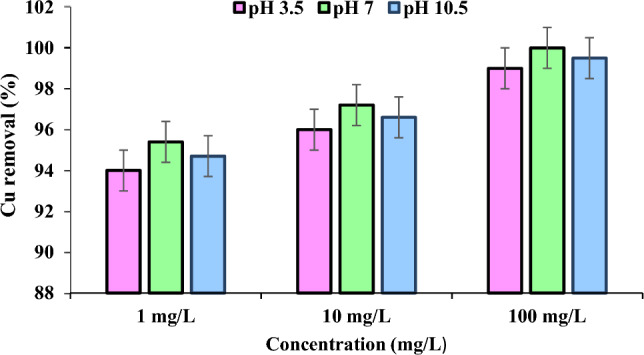
Effect of initial Cu concentration on the removal efficiency of Cu (Experimental conditions: pH: 3.5–10.5; Temperature: 20 °C; Reaction time: 30 min; Current density 2.4 mA/cm^2^).

The effect of Cu's initial concentration on the ECF process's removal efficiency is shown in Fig. 2. As evident, the removal efficiency increases as the concentration increases from 1 to 100 mg/L. In addition, as pH increases from 3.5 to 7, the removal percentage for 100 mg/L Cu concentration in the ECF reactor decreases from 99 to 100%, with a 30 min reaction time and 2.4 mA/cm^2^ current density (Table 1). Moreover, when the Cu concentration increases from 1 to 100 mg/L at a current density of 2.4 mA/cm^2^ and pH 7, the reaction time decreases from > 30 min to 30 min (Table 1).

**Table 1:** Effect of ECF on Cu removal from urban drinking water at pH 3.5–10.5

pH	Current density (mA/cm^2^)	Cu concentration 1 mg/L (%)			Cu concentration 10 mg/L (%)			Cu concentration 100 mg/L (%)		
		Time (min)			Time (min)			Time (min)		
		10	20	30	10	20	30	10	20	30
3.5	0.8	88	89.7	91.9	91	92.7	93.9	94	95.7	96.9
	1.6	90.2	93	93.6	93.2	95	95.6	96.2	98	98.6
	2.4	93.1	93.7	94	95.1	95.7	96	98.1	98.7	99
7	0.8	89.4	91.1	93.3	92.2	93.9	95.1	95	96.7	97.9
	1.6	91.9	94.4	95	94.4	96.2	96.8	97.2	99	99.6
	2.4	94.5	95.1	95.4	96.3	96.9	97.2	99.1	99.7	100
10.5	0.8	88.7	90.4	92.6	91.6	93.3	94.5	94.5	96.2	97.4
	1.6	90.9	93.7	94.3	93.8	95.6	96.2	96.7	98.5	99.1
	2.4	93.6	94.4	94.7	95.7	96.3	96.6	98.6	99.2	99.5

### Effect of water pH

**Figure 3:**
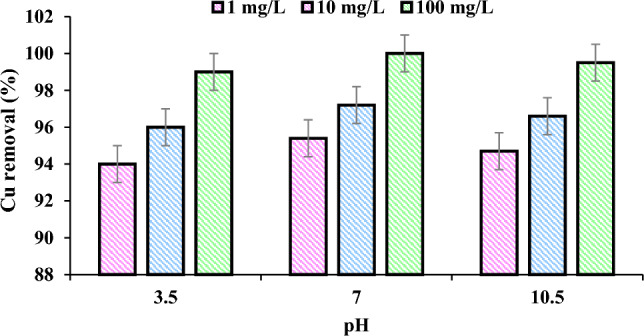
Effect of pH on the efficiency of Cu removal (Experimental conditions: Concentration: 1–100 mg/L; Temperature: 20 °C; Reaction time: 30 min; Current density 2.4 mA/cm^2^).

Initial pH values in ECF experiments ranged from 3.5 to 10.5 (Fig. 3). When the Cu concentration increased from 1 to 100 mg/L in the ECF, the mean Cu removal rose in the optimum pH 7 condition. A 100% reduction of Cu could be achieved in a batch mode with an initial concentration of 100 mg/L, a pH of 7, a reaction time of 30 min, and an anode current density of 2.4 mA/cm^2^. Increasing the pH of the solution up to 7 improved the degradation effect of this method. The optimum pH for reaching the Cu standard (1.3 mg/L) was 7.

### Effect of current density

**Figure 4:**
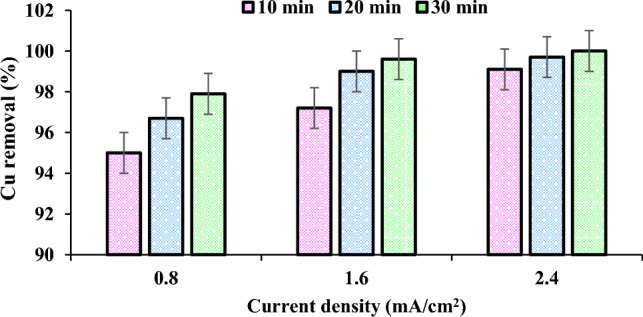
Effect of the current density of Cu removal (Experimental conditions: pH: 7; Temperature: 20 °C; Initial concentration: 100 mg/L; Reaction time: 10–30 min).

**Figure 5:**
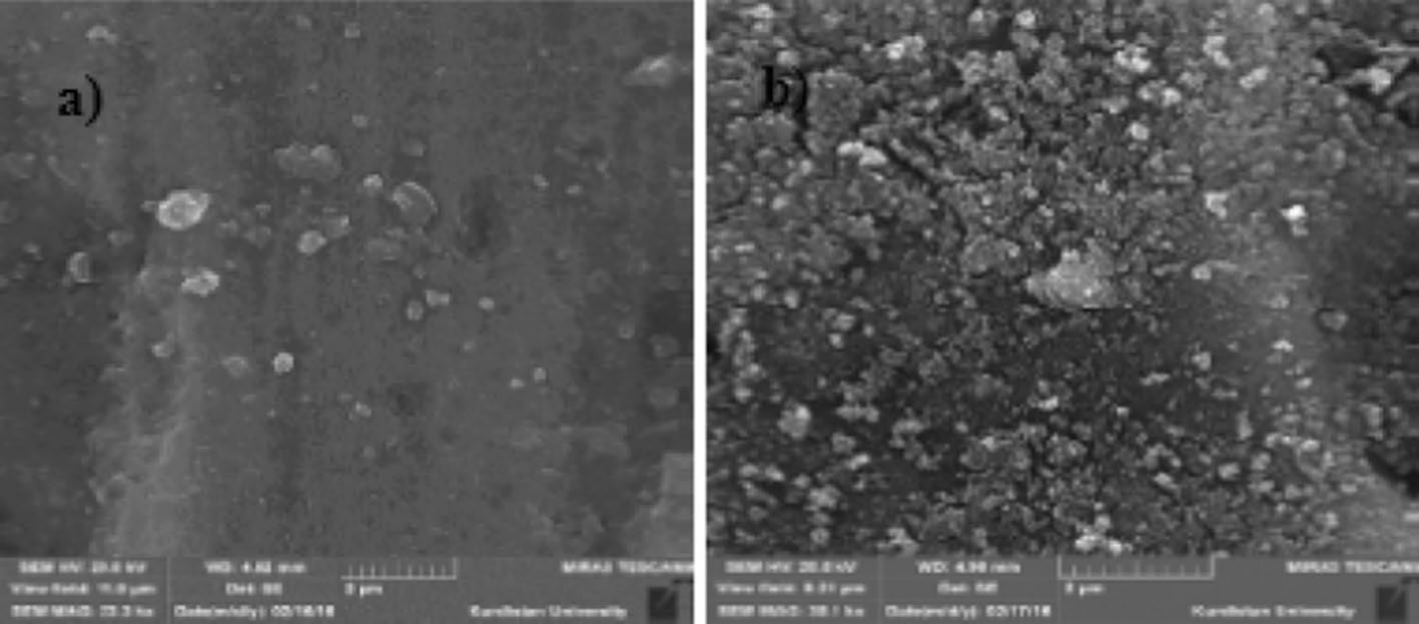
SEM images of Al electrodes a) Before; b) After (Experimental conditions: pH 7; Temperature 20 °C; Initial concentration 100 mg/L; ; Current density 2.4 mA/cm^2^; Reaction time: 30 min).

Figure 4 illustrates the current density's effect on the ECF process's removal efficiency. The optimal current density for achieving the Cu standard (1.3 mg/L) is 1.6 mA/cm^2^ at pH 7. Copper removal efficiency decreases at lower current density, reaction time, and Cu concentration (Table 1 and Fig. 4). The effect of the current density on the surface morphology of Al electrodes was studied utilizing a scanning electron microscope (SEM). As can be seen, the electrode was subjected to relatively consistent corrosion after applying an electrical current (Fig. 5).

### Optimization

**Figure 6:**
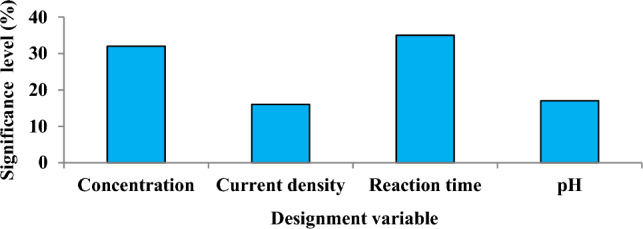
The Taguchi model (Experimental conditions: Temperature: 20 ºC; pH: 3.5–10.5; Reaction time: 0–30 min, ; Initial concentration: 1–100 mg/L; Current density 0.8–2.4 mA/cm^2^).

The Taguchi model was used to determine the optimal operational variable values. Taguchi model results for Cu removal efficiency indicate that reaction time is the most significant variable (Fig. 6).

### Kinetics

**Figure 7:**
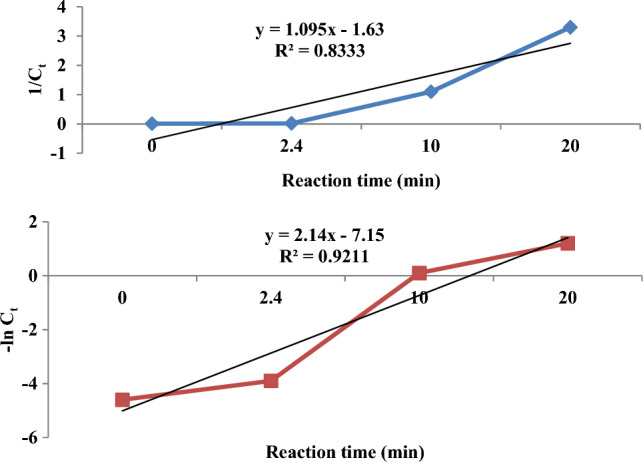
The plots of first- and second-order reaction models with the Cu removal experimental data in batch ECF reactor (Experimental conditions: Temperature: 20 ºC; pH: 7; Reaction time: 0–30 min).

The plots of the first- and second-order kinetics reaction models correspond to the Cu removal experimental data in the batch ECF reactor, indicating that the experimental data corresponds better to the second-order reaction model at pH 7 (Fig. 7). Cu's regression coefficient was calculated as R^2^ = 0.921 based on the fitted line. The apparent rate constant, K_2_, and the half-life time, t_1/2_, were calculated to be 0.29 min^−1^ and 2.4 min, respectively.

### Operational cost

According to optimal conditions (electrical current 0.03 A, electrical potential 30 V, reaction time 30 min, and water demand 40 l/day), the minimum operational cost of the ECF was calculated to be Cu concentration 100 mg/L with a removal efficiency of 100% (8857 = 160 (consumed electrode cost) + 8697 (consumed electrical energy cost)) and the maximum operational cost of the ECF was calculated to be Cu concentration 1 mg/L with a removal efficiency of 88% (9636 = 520 (consumed electrode cost) + 9116 (consumed electrical energy cost)).

## Discussion

The results indicate no correlation between Cu concentration and removal efficiency. When the Cu concentration increases from 1 to 100 mg/L at a current density of 2.4 mA/cm^2^, the reaction time of 30 min, and pH 7, the efficiency inecreases from 95.4% to 100%. Higher concentrations (100 mg/L) require less time to reduce (about 30 min), but higher initial concentrations (100 mg/L) necessitate a significant reduction in removal times (about 30 min). The efficiency starts to increase at higher concentrations at the beginning of the operation (99.1%). In contrast, the low concentration (10 mg/L) decreases the removal efficiency after the process (94.5%). This is explicable through the theory of dilute solution. Amooey et al. (2014) report comparable findings^33^. According to Hosseini et al. (2015), the removal efficiency of a concentrated solution (300 mg/L) is inferior to that of a diluted solution (2 mg/L)^34^.

The efficiency of the coagulation process can be improved by increasing the concentration of the pollutant used as a reactant in the process; doing so raises the chemical potential and, in other words, follows Luchatelier's principle. In addition, according to the collision theory, it will increase the speed. The increase in copper removal (100%) caused by an increase in current density (2.4 mA/cm^2^) is proportional to the decrease in time (about 30 min) attributable to the increased opportunity for adsorption/desorption of reactive material species and current blockage. Prasetyaningrum et al. (2021) also report increased pollutant removal due to increased concentration^35^. According to Kim et al. (2014), the removal efficiency of a concentrated solution (100 mg/L) is greater than that of dilute solutions (10 and 50 mg/L)^36^. But Moradi and Ashrafizadeh (2020) report deceasing nitrate removal due to increasing the initial concentration and increasing process duration^37^. The removal effectiveness improves as the reaction time increases. When the Cu concentration increases from 1 to 100 mg/L at a current density of 2.4 mA/cm^2^ and pH 7, the reaction time decreases from > 30 min to 30 min. This result is consistent with data previously published by Adamovic et al. (2015). They report that the highest copper removal efficiency (> 92.8%) is achieved after 5 min with an 8 mA/cm^2^ current density and aluminum electrodes^38^.

Depending on the pH, the ECF process produces different hydrogen (H_2_) concentrations from water as a flotation agent. These products play a crucial role in the ECF process for removing Cu concentrations. This effect results from the increased availability of OH^−^ anion at a lower pH, which produces more H_2_ bubbles. The decrease in Cu removal at pH 10 (99.5%) is attributable to the anode's increased oxidation of hydroxide anion. The final pH indicates a slight decrease in basicity (pH > 8) due to the precipitation of insoluble hydroxide Cu(OH)_2_. Kim et al. (2020) observed that the formation of metal hydroxide is complex at acidic pH due to the formation difficulty of OH^−^ anions^39^. Due to the formation of metal hydroxides during the ECF process, the environment's pH is optimal for removing copper. Due to the amphoteric behavior of Al(OH)_3_, acidic and basic conditions produce soluble Al^3+^ cations and Al (OH)_2_^+^ and monomeric anions Al(OH)_4_^−^, respectively. These organisms are ineffective for water purification. This result is consistent with Prasetyaningrum et al. (2019)^40^. Mota et al. (2015) observed that theoretically the pH of 8.0 is the best point for the Pb ECF during the first 20 min of flotation^41^.

Merma et al. (2020) report that monomeric anions Al(OH)_4_^−^ are the major species at pH values greater than 9.5^42^. Therefore it is concluded that the ECF reactor acts as a pH buffer. Consequently, pH adjustment is not required before treatment in practical applications. From the operational point of view, it is beneficial because there is no need to adjust the amount of pH and higher performance. According to Moussa et al. (2017), pH significantly impacts the efficiency of the EC process. Due to the high specific surface area, micro flocs of Al(OH)_3_ formed at a pH of 7, resulting in rapid Cu absorption^43^. Al has a point of zero charges (pH_PZC_) of 9.6, and Cu has an electronegativity of 1.75^44^. Therefore, the sediment charge at pH 7 is positive.

The applied current density is a key variable affecting the kinetic of Cu removal in ECF, as it regulates the amounts of anodic dissolution of electrodes acting as precipitating agents. This result is consistent with data previously published by Chen et al. (2018)^45^. With increasing current density (2.4 mA/cm^2^), the reaction time begins to decrease (about 30 min). The removal efficiency is proportional to the current density and reaction time. Due to the anode's consumption, the higher current density (2.4 mA/cm^2^) and reaction time improve the removal efficiency (100%) by increasing the formation of coagulants and bubbles in the reactor. Das and Nandi et al. (2019) report that the increased formation of coagulants due to a higher current density improves the removal efficiency^46^. Consequently, the density fluctuations of the current lead to the measurement of the Cu removal rate. pH and ORP changes are used to predict the metal forms formed in the ECF reactor. This study measures the initial and final pH and ORP values to investigate pH's and ORP's effect more effectively. The pH rises from 7.10 to 7.60 due to the formation of H_2_ gas and OH^−^ anions at the cathode electrode. The ORP decreases from 80 to -200. Majlesiet al. (2016) report that increase in current density from 0.4 to 3.2 mA/cm^2^ increased nitrate removal efficiency from 55 to 96% under optimum conditions of time and pH during the ECF^47^.

In this study, the current density increased from 0.8 to 2.4 mA/cm^2^ with a 30 min reaction time, pH 7, and 100 mg/L Cu concentration. Beyazit (2014) evidenced that 100% of Cu removal is reached at a current density of 90 mA/m^2^^48^. At a current density greater than 20 mA/cm^2^, the removal efficiency begins to decrease as the reactor temperature rises due to the disintegration of OH radicals^49^. According to Jafari et al. (2023), 90% turbidity removal is obtained at a current density of 0.0028 mA/cm^2^ after 30 min of operation. Prica et al. (2015) report that at a current density of 8 mA/cm^2^ and an operating time of 60 min, > 95% of Cu is removed^50^. The current density of 2.4 mA/cm^2^, which has an efficiency close to the optimal value while consuming less energy, was chosen for economic reasons. SEM shows the behavior of the electrodes during the process and to characterize the morphology and corrosion mechanism^51^. Based on the SEM results, systematic corrosion and indentations on the surface of the anode due to production of various aluminum hydroxides leads to forming aluminum nanostructures, which may alter the mechanism and kinetics of electron transfer and oxidation. In other hands, corrosion of the electrode over time is caused by the effect of the current density and the partial stripping of the metal ions of the aluminum electrode.

The objective of the optimization is to determine the optimal operating conditions for Cu removal from aqueous environments by the ECF process. The orthogonal array includes experimental combinations with equal probability and controllable factors^52^. The Taguchi model results for Cu removal efficiency indicate that reaction time (35%) is the most significant variable. According to Apaydin et al. (2020), the contributions of pH, current density, and reaction time to COD removal efficiency are, respectively, 79.35%, 6.45%, and 4.46%^53^. According to Ozyonar et al. (2017), current density contributes the least to color removal efficiency^54^. Kurtoglu Akkaya and Oden (2022) reported that reaction time and current density contribute the most to 4-chlorophenol removal efficiency^55^. Kashi (2017) reported that reaction time the most to was to phenanthrene removal efficiency^56^.

Vrsalovic et al. (2023) state that adding zeolite is the most effective factor for chemical oxygen demand removal^57^. The apparent rate constant, K_2,_ and the half-life time, t_1/2_, are calculated to be 0.29 min^−1^ and 2.4 min, respectively. This result contradicts the EC experiments Mohammadlou et al. (2014) performed with iron-steel electrodes. They concluded that the CR degraded according to a first-order kinetic model^58^. Selim et al. (2017) concluded that the heavey metals incuding Cu^+2^ degraded according to a first-order kinetic model^59^.

Al^+3^ cations react with H_2_O and OH^−^ anions to produce coagulant agents in the reactor during the ECF process, such as a monomeric (Al(OH)_2_^+^, Al(OH)_2_^2+^, Al_2_(OH)_2_^4+^, Al(OH)_4_^−^) and polymeric hydroxides (Al_6_(OH)_15_^3+^, Al_7_(OH)_17_^4+^, Al_8_(OH)_20_^4+^, Al_13_O_4_(OH)_24_^7+^, Al_13_(OH)_34_^5+^). The significant adsorption capacity of these agents is one of their distinguishing characteristics. Consequently, these agents can form bonds with Cu during the ECF process. This result is consistent with data previously published by Khue et al. (2014)^60^. Cu removal in the ECF reactor is concluded to be related to redox and adsorption. Suspended aluminum hydroxides are capable of removing Cu from water through adsorption, co-precipitation, redox, and electrostatic attraction, followed by a coagulation/flotation process. In addition to hydroxide precipitation and absorption on the Al(OH)_3_, Al(OH)_2_^+^, and Al(OH)^+^ flocs, from the perspective of Cu, Cu is reduced via direct electro-reduction at the cathode and electrolysis deposition. Equation (14) illustrates the removal mechanisms:

14 $${\text{3Cu}}^{{{2} + }} + {\text{ 2Al}}\to{\text{2Al}}^{{{3} + }} + {\text{ 3Cu}}$$

Cu produced from the reaction is removed from water due to adsorbing on the aluminum cathode surface and precipitating with coagulants. Consequently, as concentrations increase (100 mg/L), operational expenses begin to diminish (8857). The current efficiency, a significant variable in the ECF reactor, is 190.6%. This result is consistent with data previously published by Kobya et al. (2010). Kobya et al. demonstrate that the calculated cost of treatment with an EC reactor is $1.05 m^−3^ for cadmium and $2.45 m^−3^ for nickel and cyanide^61^. Shah et al. (2017) demonstrate that calcuated cost of the experimental design about 4.486 US$/dm^3^^62^.

## Conclusions

This study examined the reduction of copper from urbane drinking water in an electrocoagulation/flotation reactor in a batch with monopolar aluminum electrodes connection mode. To this end, various operational factors' effects on process reduction efficiency were investigated. The tests yielded the following results:

- Cu reduction is influenced by Cu concentration. According to the collision theory, increasing the concentration from 1 to 100 mg/L increases the removal efficiency.
- Due to the formation of hydrogen (H_2_) from water as a flotation agent, the degradation effect of this method is highly pH-dependent and enhanced by a pH increase of up to 7.
- The optimum current density for reaching the Cu standard (1.3 mg/L) is 1.6 mA/cm^2^. By increasing the formation of coagulants and bubbles, a greater current density improves the removal efficiency.
- Operating time significantly affects Cu reduction throughout the process; for a given set of experimental conditions, Cu reduction improves with increasing reaction time.
- Cu reduction during reactor operation follows a second-order rate equation.
- The removal process is linked to the cathode's increasing reduction conditions due to hydrogen evolution. Cu is reduced via direct cathodic electroreduction and electrolysis deposition.
- As Cu concentration increases, operational costs decrease. At higher concentrations, the trend slows down.
- The fact that ECF proposes advanced technologies for water and wastewater treatment. However, this process is not yet fully optimized, and more research is required before it can be implemented globally as a safe water purification method. Among the research's strengths and innovations are investigating the cost of the process, presenting the mechanism, determining the level of importance of the variables affecting the removal efficiency, SEM of the electrode before and after the process, and reaction kinetics. Among the limitations of this research is the failure to investigate the removal efficiency of the ECF reactor in the presence of chemical variables of water quality; therefore, it is suggested that future studies investigate the effect of these variables on the removal efficiency and the intensification effect of the continuous and batch ECF reactor with other types of electrodes.
- ECF is an efficient and practical method for achieving a high degree of Cu reduction from drinking water in batch and monopolar electrode connection mode and a promising method for treating Cu-contaminated drinking water.

## Data Availability

The datasets generated and analyzed during the current study were available from the corresponding author on reasonable request.
